# Change of the duodenal mucosa-associated microbiota is related to intestinal metaplasia

**DOI:** 10.1186/s12866-019-1666-5

**Published:** 2019-12-09

**Authors:** Jian Gong, Lixiang Li, Xiuli Zuo, Yanqing Li

**Affiliations:** 1 Department of Gastroenterology, Qilu Hospital, Shandong University, Jinan, 250012 China; 2 Department of Gastroenterology, Taian City Central Hospital, Taian, Shandong Province China; 3 Laboratory of Translational Gastroenterology, Qilu Hospital, Shandong University, No. 107, Wenhuaxi Road, Jinan, 250012 China

**Keywords:** Intestinal metaplasia, Duodenal microbiota, Dysbiosis, *Helicobacter pylori*

## Abstract

**Background:**

In this study, we aimed to investigate the characteristics of the duodenal mucosal microbiota of patients with intestinal metaplasia (IM) and compare it with those of the gastric mucosal microbiota.

**Method:**

We collected the duodenal and gastric mucosal samples from 10 adult patients with IM and 10 healthy controls (HC). The V3-V4 region of the bacterial 16S rRNA gene was examined by high throughput sequencing method.

**Results:**

The diversity of the HC duodenal microbiota was higher than that of IM patient based on the Shannon and Simpson index while the Chao indices of IM duodenal mucosal microbiota was significantly higher than that of gastric mucosal microbiota of patients with IM. There was a marked difference in the duodenal microbiota structure between patients with IM and HC (ANOSIM, R = 1, *P* = 0.001). We also found that the *Helicobacter pylori* infection in gastric mucosa did not influence the structure of duodenal mucosal microbiota. The gastric mucosal microbiota structure significantly differed between patients with IM and HC who were *H. pylori*-negative (ANOSIM, R = 0.452, *P* = 0.042) or *H. pylori*-positive (ANOSIM, R = 0.548, *P* = 0.003), respectively. For duodenal mucosal microbiota, genera *Lactococcus*, *Flavobacterium*, *Psychrobacter*, *Mysroides*, *Enhydrobacter*, *Streptococcus*, and *Leuconostoc* were enriched in patients with IM. In contrast, genera *Bacillus*, *Solibacillus*, *Lysinibacillus*, *Exiguobacterium*, *Oceanobacillus*, and *Paenibacillus* were enriched in HC.

**Conclusion:**

A marked dysbiosis duodenal mucosal microbiota in patients with IM was observed, and this dysbiosis might be responsible for IM pathogenesis.

## Background

Recently, gastric cancer (GC) has been reported as the fourth most common malignancy and one of the leading causes of cancer-related deaths worldwide. It has a particularly high incidence in East Asia, Eastern Europe, and Central and South America [1]. Gastric carcinogenesis has been hypothesized as a multistep process comprising superficial gastritis (SG), chronic gastritis, atrophic gastritis (AG), intestinal metaplasia (IM), dysplasia, and then carcinoma [2]. IM is a crucial risk factor for GC and is considered part of the pathologic spectrum of gastric mucosal atrophy [3]. According to epidemiological evidence, IM condition may be reversed following treatment with antioxidant agents for eradicating *Helicobacter pylori* [4]. However, IM is still believed to be the “point of no return” during the histological process ranging from chronic gastritis to cancer [5]. Thus, it is crucial to explore the molecular mechanisms underlying IM pathogenesis and develop strategies to interfere with the gastric carcinogenesis.

Recent studies show that microbial changes are related to the histological stages of gastric tumorigenesis. Chronic *H. pylori* infection can cause mucosal inflammation and induce histological change. It is also recognized as a major risk factor for GC. Nevertheless, only 3% of *H. pylori*-infected patients develop GC [6]. Moreover, it was found that *H. pylori* is usually undetectable in gastric cancer samples [7]. These studies suggest that *H. pylori* infection might only be an early event for the gastric mucosa which would further undergo oncogenic changes, and indicate the potential role of mucosal microbes, with the exception of *H. pylori*, in gastric carcinogenesis. The dominant phylum in mucosal microbes was Proteobacteria in both *H. pylori*-negative and *H. pylori*-positive samples [8]. Two previous studies demonstrated that the microbiota of patients with IM was found to partially overlap with the gastritis and cancer group among patients with *H. pylori* infection [9, 10]. Li et al. (2017) found that the microbiota of gastritis samples mostly overlapped with that of IM samples. In contrast, microbiota of patients with IM and GC had significantly low microbial richness, while the β-diversity of microbiota of SG, AG and IM was similar in overall differences, with the exception of that of GC [11]. These conflicting results suggest that IM might be the key point in microbiota change and there might be other potential factors involved in gastric tumorigenesis, especially in patients with IM.

Most of the studies on gastric cancer have focused on gastric microbiota dysbiosis. Recent evidence has revealed that the small intestinal microbiota, especially the mucosal microbiota, might play a crucial role in gastrointestinal health [12]. Dysbiosis of the small intestinal microbiota has been found in celiac disease [13], chronic liver disease [14], diabetes mellitus [15], and irritable bowel syndrome [16]. However, the information regarding the role of duodenal microbiota in IM is still limited.

In this study, we investigated the mucosal microbiota of the duodenum and stomach in patients with IM and compared it with those of HC.

## Results

### Participants

A total of 20 participants, including 10 IM (6 males, 4 females, 6 HP-positive) and 10 healthy individuals as control (5 males, 5 females, 4 HP-positive) were recruited in this study (Table 1 and Additional file 1: Table S1). No significant differences in gender (male: 60.0% vs. 50.0%, *P* = 0.65) and age (51.3 ± 8.01 vs. 57.80 ± 7.22, *P* = 0.07) were detected between the IM and HC groups, respectively.

**Table 1:** Characteristics of the study participants

	IM (*n* = 10)	HC (*n* = 10)	*P*
Sex (male, %)	6 (60%)	5 (50%)	0.65
Age	57.8 ± 7.22	51.30 ± 8.01	0.31
HP+ (16 s RNA sequencing)	6	4	0.37
HP+ (RUT)	6	4	0.37

*RUT* rapid biopsy urease test

### Small intestinal bacterial diversity is lower in patients with IM

**Fig. 1:**
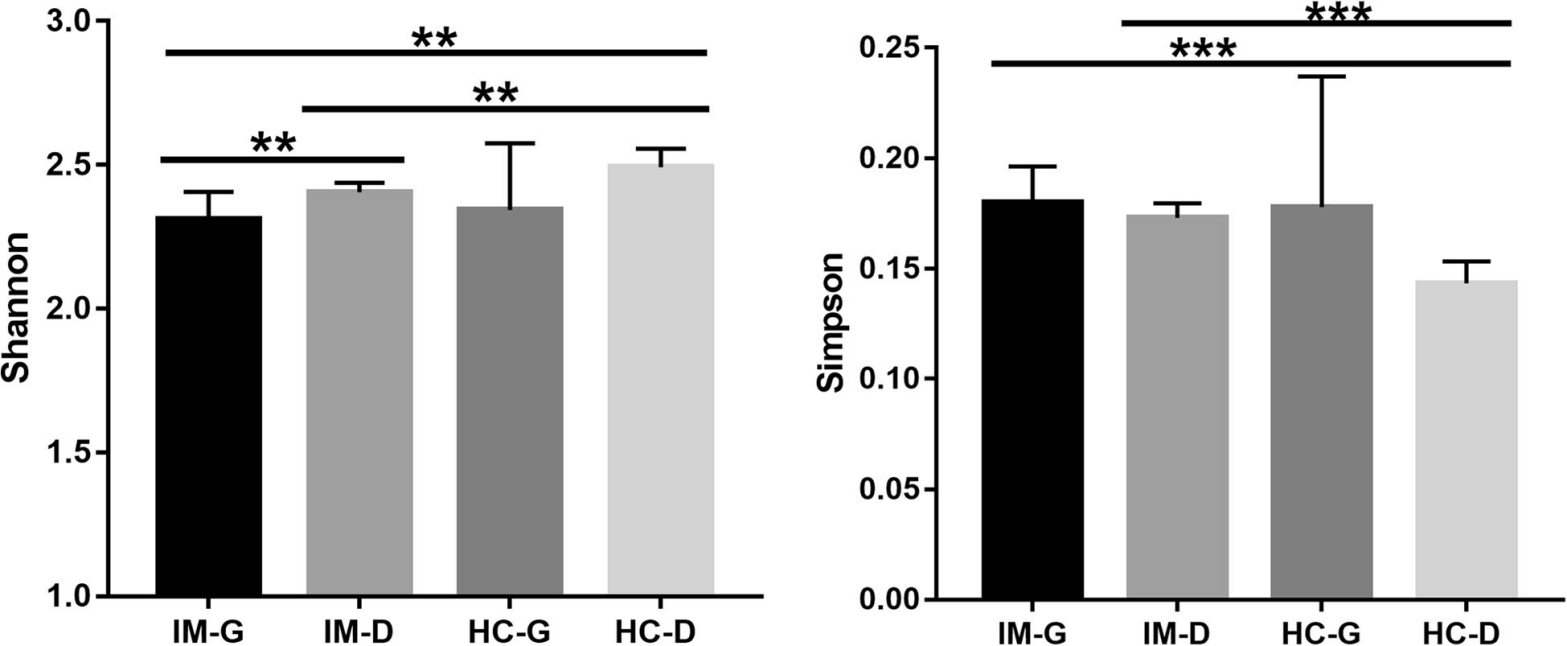
The α-diversity of the gut microbiota in HSP and control. Unpaired t-test were used for comparing the Ace and Shannon index. *, *P*<0.05; **, *P*<0.01, ***, *P*<0.001

To detect the microbiota dysbiosis associated with IM, the microbial diversity and richness of gastric and duodenal mucosal biopsy samples were estimated by analyzing of hypervariable V3-V4 regions of the 16S ribosomal RNA gene. An average of 37,165 high quality sequences per sample was obtained after quality-filtering steps. The estimate of coverage reached > 99.9% for all samples. After removing the rare microbial OTUs, 27,698 sequences per sample and 125 OTUs were obtained for further analysis. Next, we estimated the α-diversity of the microbiota (Additional file 1: Table S1) and compared the mean values between groups. The results are shown in Fig. 1. Compared with IMG and IMD, microbiota of HC-G and HC-D had slightly reduced Chao1-estimated microbial richness with no statistical differences (data not shown). However, the diversity of HC-D was higher than that of IM-D based on the Shannon and Simpson indices. Meanwhile, the two Chao indices of duodenal mucosal microbiota were higher than those of gastric mucosa and only the Chao index of IM duodenal mucosal microbiota was significantly higher than that of IM-G (Wilcoxon rank-sum test, *P*<0.05).

### Gastric and duodenal microbiota structure is altered in patients with IM

**Fig. 2:**
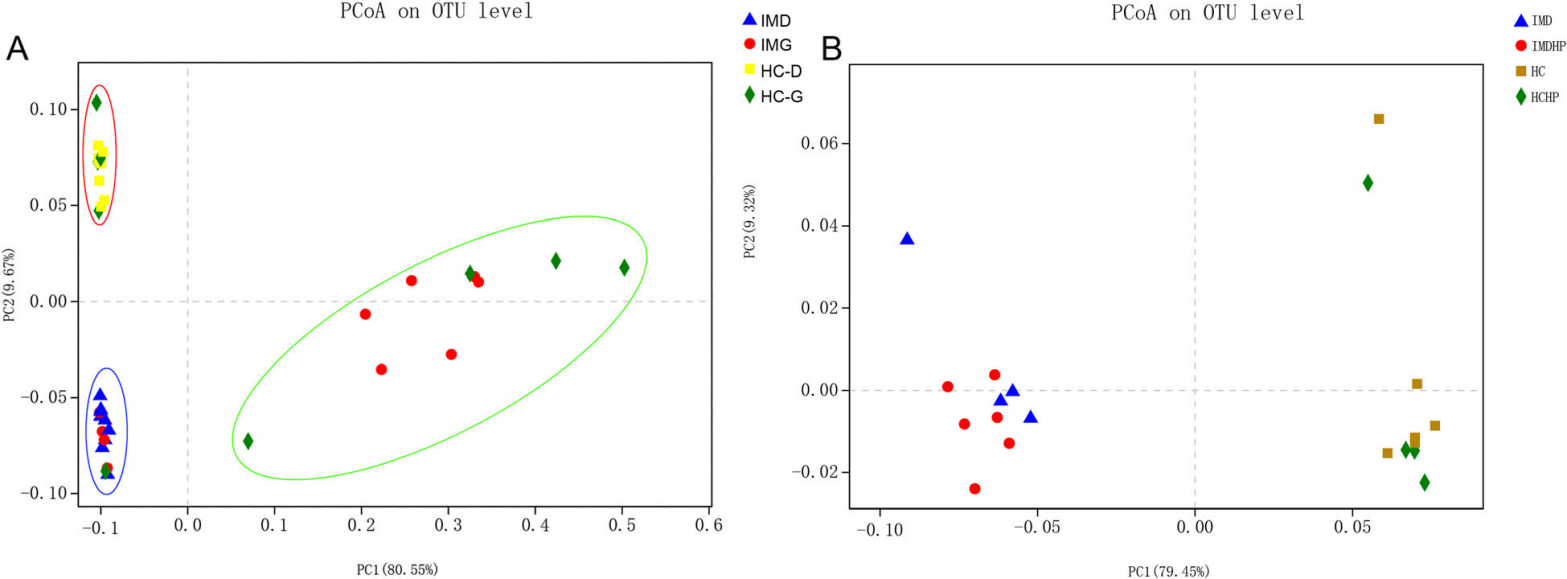
PCoA analysis of the microbiota among IM and HC. **a**, Comparison of the duodenal and gastric microbiota. Green circle, gastric samples with HP infection; red circle, duodenal and gastric sample of HC; blue circle, duodenal and gastric sample of IM. HCD, duodenal samples of HC; HCG, gastric sample of HC; IMG, gastric sample of HC; IMD, duodenal samples of IM. **b**, The influence of HP infection in duodenal microbiota. IMD duodenal samples of IM without HP infection; IMDHP, duodenal samples of IM with HP infection; HC, duodenal samples of HC without HP infection; HCHP, duodenal samples of HC with HP infection

The similarity of the bacterial community structures between patients with IM and HC was evaluated by PCoA (Fig. 2a). For duodenal mucosal microbiota, significant differences were observed in the microbiota structure between IMD and HC-D (ANOSIM, R = 1, *P* = 0.001). We also found that the HP infection in gastric mucosa did not influence the structure of duodenal mucosa microbiota (Fig. 2b). The gastric mucosal microbiota structure significantly differed between IMG and HC-G in HP-negative patients (ANOSIM, R = 0.452, *P* = 0.042) or HP-positive patients (ANOSIM, R = 0.548, *P* = 0.003), respectively. There was no significant difference between IMG (HP-) and IMD (ANOSIM, R = 0.37, *P* = 0.05), as well as between HC-G (HP-) and HC-D (ANOSIM, R = 0.176, *P* = 0.075).

### Duodenal mucosa microbiota composition is altered in patients with IM

**Fig. 3:**
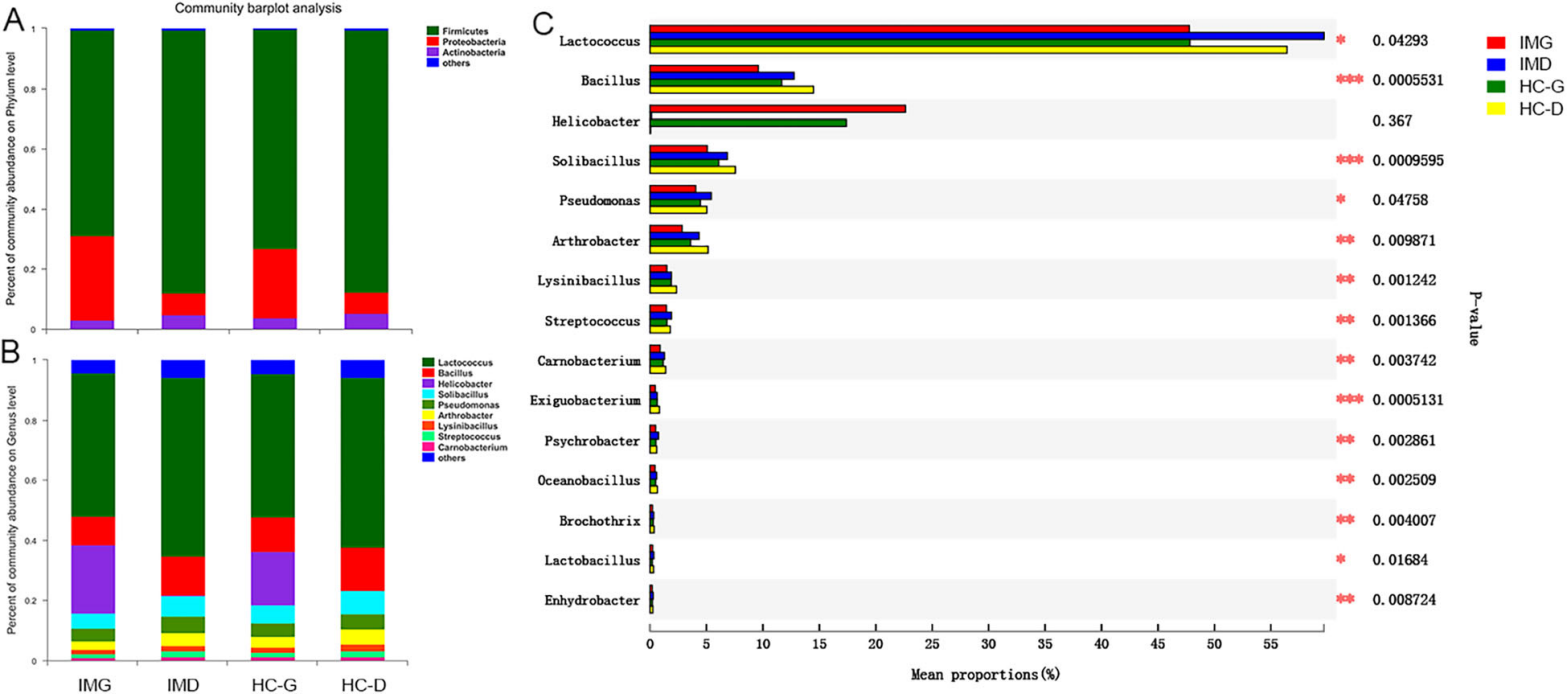
The relative taxa abundance between IM and HC. **a**, relative taxa abundance in phylum level; **b**, relative taxa abundance genus level; **c**, comparison of relative taxa abundance of genus level

**Fig. 4:**
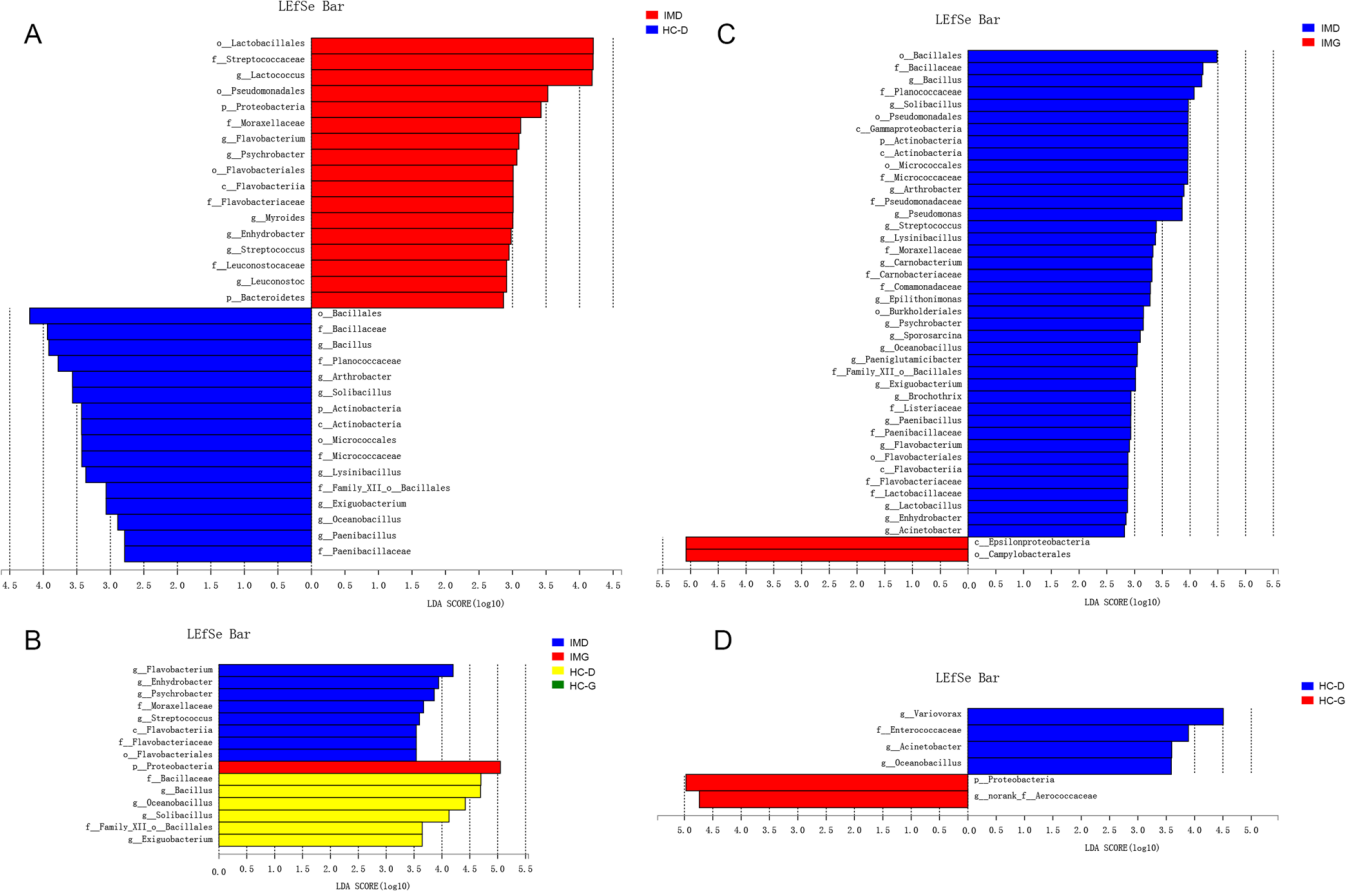
The most differentially abundant taxa between IM and HC based on LEfSe analysis. **a** The most differentially abundant taxa between IM and HC in duodenal microbiota. **b** The most differentially abundant taxa between IM and HC in duodenal and gastric microbiota. **c** The most differentially abundant taxa between duodenal and gastric microbiota in IM patients. **d** The most differentially abundant taxa between duodenal and gastric microbiota in HC

Most of the gastric and duodenal mucosal bacteria detected in this study belong to the following three phyla: Firmicutes, Proteobacteria, and Actinobacteria (Fig. 3a). The main nine genera of gut microbiota (percentages were above 1%) comprised up to 90% of the total microbiota and included the following: *Lactococcus*, *Bacillus*, *Helicobacter*, *Solibacillus*, *Pseudomonas*, *Arthrobacter*, *Lysinibacillus*, and *Streptococcus* (Fig. 3b). We compared the proportions of dominant genera and found that most of them changed as shown in Fig. 3c. The specific taxa that most likely contributed to the differences between IM and HC group were revealed by linear discriminant analysis of effect size (Fig. 4). In duodenal mucosal microbiota, the genera *Lactococcus*, *Flavobacterium*, *Psychrobacter*, *Mysroides*, *Enhydrobacter*, *Streptococcus*, and *Leuconostoc* was found to be enriched in patients with IM. In contrast, the genera *Bacillus*, *Solibacillus*, *Lysinibacillus*, *Exiguobacterium*, *Oceanobacillus*, and *Paenibacillus* were enriched in HC (Fig. 4a). However, there were no significant specific taxa in gastric mucosa microbiota between IM and HC (data not shown). There was also a greater number of specific taxa between gastric and duodenal mucosa microbiota in patients with IM than HC. Eighteen genera including *Bacillus*, *Solibacillus*, and *Arthrobacter* were enhanced in duodenal mucosal microbiota of patients with IM and only three genera, including *Variovorax*, *Acinetobacter*, and *Oceanobacillus*, were enhanced in the duodenal mucosa microbiota of HC (Fig. 4c and d). When the microbiota of four groups was compared, four genera including *Flavobacterium Enhydrobacter*, Psychrobacter, and *Streptococcus* were found to be enriched in the duodenal mucosal microbiota of patients with IM, while the genera *Bacillus*, *Oceanobacillus*, *Solibacillus*, and *Exiguobacterium* were enriched in duodenal mucosal microbiota of HC (Fig. 4b). There was no specific genus enriched in the gastric mucosa microbiota of patients with IM and HC.

## Discussion

In this study, microbial communities in the duodenal mucosa of patients with IM showed significant differences with those of HC, including a lower diversity, different microbiota structure and specific taxa. We also found that the gastric mucosal microbiota of HC was similar to their duodenal mucosal microbiota. In contrast, the gastric mucosal microbiota of patients with IM differed from their duodenal mucosal microbiota. These data indicated a potential role for duodenum microbiota in IM pathology.

Microbiota dysbiosis has been detected in many gastrointestinal and systemic diseases including IM and gastric cancers. However, the changes in gastric microbiome compositions including microbial diversity and richness across stages of gastric carcinogenesis are inconsistent in different studies [8–11]. It has been previously reported that the diversity, evenness and overall composition was similar between patients with non-atrophic gastritis and patients with IM [7, 9, 10] [7]. In contrast, Li et al. (2017) found that the normal group had higher Shannon and phylogenetic diversity indices than those of IM (*P* = 0.009) [8]. Meanwhile, Coker et al. (2018) found that microbiomes of IM had significantly reduced Chao1-estimated microbial richness compared with that of superficial gastritis. However, there was no significant difference among superficial gastritis, atrophic gastritis and IM based on the evaluation of the overall differences in β-diversity [11]. In the current study, we found that the diversity of HC-D was higher than that of IM-D based on the Shannon and Simpson indices, while the gastric mucosal microbiota structure of patients with IM differed from that of HC either with or without *H. pylori* infection, respectively (Fig. 2). The contradiction may be partially caused by different variables which could affect the gut microbiome composition including gender, age, diet and *H. pylori* infection. Further studies focusing on the distribution of gastric microbiota in the development of GC are still needed.

The duodenal mucosal microbiota has garnered considerable attention recently. It has been reported that small intestinal microbiota dysbiosis with an abundance of *Proteobacteria* influence celiac disease pathogenesis [17]. Li et al. (2017) found that the mucosal microbiota of duodenal samples differed from that of rectal samples in HC; additionally, this difference has been found to be less pronounced in IBS-D. Concurrently, the number of shared OTUs and genera of duodenal rectal samples in IBS-D was more than those of HC. These authors suggested that the shared mucosal-associated microbiota of duodenum and rectum may contribute to the etiology and pathophysiology of IBS-D [8]. It has also been found that the duodenal microbiota of obese individuals displays an alteration in fatty acid and sucrose breakdown pathways possibly induced by diet imbalance [18]. In symptomatic gastritis patients, the patient appraisal of gastrointestinal disorders symptom severity index demonstrated a stronger relation with the duodenal microbiota than with the gastric microbiota. Meanwhile, the combined inflammation score was inversely related with the abundances of *S. epidermidis* (r = 0.346) and *M. osloensis* (r = 0.305) in the duodenum [19]. These results indicated that the small intestinal microbiota is an important modulator of health. In this study, we found that the diversity and structure of duodenal mucosal microbiota of patients with IM were significantly different from those of HC (Fig. 1 and Fig. 2). The results also demonstrate that the HP infection in gastric mucosa had no influence on the structure of the duodenal microbiota (Fig. 2b). Additionally, there were no significant difference between IMG (HP-) and IMD (ANOSIM, R = 0.37, *P* = 0.05), as well as between HC-G (HP-) and HC-D (ANOSIM, R = 0.176, *P* = 0.075). Although the sample size was small, these results still suggest that the duodenal microbiota might play a potential role in the pathogenesis of IM, especially in HP negative patients. In future, larger, multicenter studies are needed to explore the role of duodenal microbiota in IM pathogenesis in the future.

As duodenal microbiota dysbiosis was found only in patients with IM, it might be a target for treatment of IM. In recent year, the probiotics have been used to treat many diseases based on modulation the gut microbiota [19–21]. The concentration of living bacteria in commercial probiotics products is much higher as than in the duodenal flora (10^9^ vs 10^5^ microbes/mL, respectively). Consequently, probiotic intake may have a greater influence on the duodenal microbiota than on the distal gut microbiota [22]. Probiotics have also been used for treatment of small intestinal bacterial overgrowth in children [23]. Further studies on modulation of the duodenal microbiota for IM treatment through microbiota-modulating therapies, such as probiotics, are needed.

## Conclusion

Duodenal microbiota dysbiosis was found in patients with IM. This dysbiosis might play a role in the pathogenesis of IM and serve as a potential therapeutic target for the condition.

## Methods

### Study population

Patients scheduled for gastroscopy examination were enrolled in this study at Qilu Hospital, Shandong University, according to the inclusion and exclusion criteria. The inclusion criteria were as follows: (i) dyspeptic symptoms and older than 40 years; (ii) *H. pylori* infection, IM or AG verified by histological results. The exclusion criteria were as follows: (i) presence of gastrectomy, acute gastrointestinal bleeding, or gastric neoplasia; (ii) presence of conditions unsuitable for the performance of a gastroscopy, such as coagulopathy, impaired renal function (creatinine level > 1.2 mg/dL), breastfeeding or pregnancy; (iii) people who did not provide informed consent. In addition, 10 healthy volunteers were examined to ensure that they had no gastritis, metabolic, cardiovascular or cerebrovascular diseases, or cancer and selected as the control group. All volunteers enrolled in this study were not administered pharmacological agents (such as antibiotics, laxatives, antidiarrheal agents, and even antidepressants) or probiotic supplements for at least four weeks prior to the study. This study was approved by the Clinical Ethics Committee of Shandong University Qilu Hospital. All patients and HC received information concerning their participation in the study and provided written informed consent.

### Mucosal sample collection, DNA extraction, and pyrosequencing

The duodenal and gastric mucosal biopsy specimens of patients with IM and HC were collected. The samples were immediately stored at − 80 °C and then shipped to Majorbio (Shanghai, China) for high throughput sequencing. FastDNA SPIN kit (MP Biomedicals, California, USA) was used to extract DNA. PCR (ABI GeneAmp 9700, ABI, USA) amplified the V3-V4 region of the bacterial 16S rRNA gene using the primers 338F (ACTCCTACGGGAGGCAGCAG), and 806R(ACTCCTACGGGAGGCAGCAG), and the TransStartFastPfu DNA Polymerase (TransGen, Beijing, China). Next, the amplicons were purified using gel extraction (AxyPrep DNA GelExtraction Kit, Axygen, California, USA) and quantified using QuantiFluor-ST (Promega, USA). The purified products were pooled to an equimolar concentration, and sequenced using an Illumina MiSeqsystem (Illumina, California, USA) according to standard protocols.

### Taxonomy quantification using 16S rRNA gene sequences

Raw FASTQ data were demultiplexed and quality-filtered using Trimmomatic and then merged using FLASH according to the following criteria: (i) all reads were deleted at any site achieving an average quality score less than 20 over a 50-bp sliding window. (ii) primers were accurately matched permitting two nucleotide mismatching, and all the reads containing ambiguous bases were eliminated. (iii) Sequences whose overlap was longer than 10-bp were merged by the overlap sequence.

The data analysis was performed on the open cloud platform of Majorbio (www.i-sanger.com). The operational taxonomic units (OTUs) were clustered with the similarity cutoff of 97% by UPARSE (version 7.1, http://drive5.com/uparse/), while chimeric sequences were detected and eliminated by UCHIME. The taxonomy of each 16S rRNA gene sequence was analyzed by the RDP Classifier algorithm compared to the Silva (SSU128) 16S rRNA database with a confidence threshold of 70% (http://rdp.cme.msu.edu/).

The abundance of OTUs was normalized by a standard of sequence number according to the least sequences of the samples. Subsequent analysis of α-diversity and β-diversity, principal coordinate analysis (PCoA), were executed by QIIME with these output normalized data. Linear discriminant analysis (LDA) effect size (LEfSe) analyses were executed using the LEfSe tool. Analysis of similarity test (ANOSIM) was carried out with PRIMER 6 software package (PRIMER-E Ltd., Luton, UK) to compare the differences of microbial communities between the patients with IM and HC.

### Statistic analyses

Data are presented as the mean ± SD. The normality of the distribution was contrasted demonstrated with the Kolmogorov-Smirnov test for normality. The Chi-square test was performed to evaluate the effects of gender. Continuous variables were compared with independent sample and unpaired-samples t-tests. *P* values < 0.05 were considered as statistically significant. Analyses were carried out using the SPSS statistical package, version 24.0 (SPSS).

## Supplementary information

**Additional file 1: Table S1.** The Hp status and α-diversity of each sample.

## Data Availability

The raw data are available from the SRA database (SRP224905).

## Abbreviations

GC: Gastric cancer
HC: Healthy control
IM: Intestinal metaplasia
OTU: Operational taxonomic unit
