# Supplementary material for: Change of the duodenal mucosa-associated microbiota is related to intestinal metaplasia
Source: BMC Microbiol. 2019 Dec 9;19:275. doi: 10.1186/s12866-019-1666-5 (PMC6900849; doi:10.1186/s12866-019-1666-5)
Supplement: Supplementary file 1 — Additional file 1: Table S1. The Hp status and α-diversity of each sample. [file 12866_2019_1666_MOESM1_ESM.docx]

Table S1 The Hp status and α-diversity of each sample.

| Sample ID | Hp | Sample location | Shannon | Simpson |
| --- | --- | --- | --- | --- |
| HC1 | - | D | 2.52 | 0.14 |
|  |  | G | 2.53 | 0.14 |
| HC10 | + | D | 2.51 | 0.14 |
|  |  | G | 2.22 | 0.17 |
| HC2 | - | D | 2.46 | 0.15 |
|  |  | G | 2.51 | 0.14 |
| HC3 | - | D | 2.51 | 0.14 |
|  |  | G | 2.48 | 0.14 |
| HC4 | + | D | 2.34 | 0.17 |
|  |  | G | 2.50 | 0.14 |
| HC5 | - | D | 2.49 | 0.14 |
|  |  | G | 2.54 | 0.13 |
| HC6 | + | D | 2.57 | 0.14 |
|  |  | G | 1.95 | 0.28 |
| HC7 | + | D | 2.55 | 0.14 |
|  |  | G | 1.94 | 0.29 |
| HC8 | - | D | 2.46 | 0.15 |
|  |  | G | 2.40 | 0.16 |
| HC9 | - | D | 2.50 | 0.14 |
|  |  | G | 2.35 | 0.19 |
| IM1 | - | D | 2.37 | 0.18 |
|  |  | G | 2.28 | 0.20 |
| IM10 | + | D | 2.42 | 0.17 |
|  |  | G | 2.22 | 0.20 |
| IM2 | - | D | 2.42 | 0.17 |
|  |  | G | 2.33 | 0.19 |
| IM3 | + | D | 2.40 | 0.18 |
|  |  | G | 2.23 | 0.18 |
| IM4 | + | D | 2.47 | 0.17 |
|  |  | G | 2.47 | 0.15 |
| IM5 | + | D | 2.35 | 0.18 |
|  |  | G | 2.14 | 0.19 |
| IM6 | + | D | 2.40 | 0.17 |
|  |  | G | 2.33 | 0.18 |
| IM7 | + | D | 2.40 | 0.17 |
|  |  | G | 2.35 | 0.16 |
| IM8 | - | D | 2.43 | 0.16 |
|  |  | G | 2.38 | 0.17 |
| IM9 | - | D | 2.39 | 0.17 |
|  |  | G | 2.38 | 0.18 |

D, duodenal sample; G, gastric sample
